# The Interplay of Malignancy and Endocarditis: A Report of a Rare Case of Marantic Endocarditis in Metastatic Lung Adenocarcinoma

**DOI:** 10.7759/cureus.63456

**Published:** 2024-06-29

**Authors:** Nehemias Guevara, Jorge Sanchez, Volha Chapiolkina, Esmirna M Perez Rosario, Maria C Tole, Yemesrach Mekonen, Noemy Coreas, Ilmana Fulger

**Affiliations:** 1 Internal Medicine, St. Barnabas Hospital Health System, New York, USA; 2 Gynecologic Oncology, National University of El Salvador, San Salvador, SLV; 3 Hematology-Oncology, St. Barnabas Hospital Health System, New York, USA

**Keywords:** metastatic non-small cell lung cancer, systemic emboli, marantic endocarditis, adenocarcinoma lung, hematology

## Abstract

Endocarditis involves inflammation of the inner layer of the heart, known as the endocardium. This condition typically presents with vegetation, with bacteria and fungi usually being the primary culprits. It is divided into two main categories based on its cause: infectious endocarditis and noninfectious endocarditis (NIE). Most cases of NIE are associated with malignancies, most of which are adenocarcinomas of the pancreas and lungs. We present the case of a 63-year-old man with recently diagnosed stage 3 non-small cell lung cancer and a previous history of thrombosis to the renal and popliteal arteries alongside an extensive cardiovascular history who presented with blurry vision secondary to multiple acute ischemic strokes, initially thought to be a consequence of septic emboli due to bacterial endocarditis; however, further workup revealed otherwise, illustrating the complex relationship between malignancy and endocarditis and its ramification.

## Introduction

Endocarditis is an inflammation of the endocardium, the inner layer of the heart. It is characterized by vegetation, with bacteria and fungi being the most common causes. It is classified as infectious endocarditis (IE) or noninfectious endocarditis (NIE) [1,2].

IE is common in degenerative valve diseases, prosthetic valves, indwelling catheters, implanted cardiac devices, diabetes, immunosuppression, and chronic heart disease. Rheumatic heart disease remains a significant risk factor in the developing world [3,4].

The actual incidence of NIE is still unknown; extensive necropsy reports have shown an incidence of 3.7% [1,5,6]. Previous studies have shown that it is an underdiagnosed entity; Bussani et al. analyzed more than tens of thousands of autopsies, and none of the NIEs were diagnosed premortem [5].

NIE is associated with autoimmune diseases, such as systemic lupus erythematosus (SLE), rheumatoid arthritis (RA), and other rare immune disorders (granulomatosis with polyangiitis, Behçet’s disease (BD), and Still’s disease of adulthood) [7-11], as well as HIV patients [12]. However, most cases are associated with malignancies, most of which are adenocarcinomas of the pancreas and lungs.

IE and NIE have different symptoms. Most patients with NIE are not investigated until they have experienced an acute stroke because they usually lack systemic infectious characteristics such as fever [1,5].

We present the case of a 63-year-old man with metastatic lung adenocarcinoma and hepatitis B refractory to treatment with an extensive cardiovascular history who presented with multiple acute ischemic strokes.

## Case presentation

A 63-year-old man with a medical history including hypertension, coronary artery disease, untreated HIV, and recently diagnosed lung adenocarcinoma presented to the ED with sudden onset blurriness in his right eye, accompanied by severe bilateral frontotemporal headache and left-sided pleuritic chest pain with associated shortness of breath. He had a history of deep venous thrombosis, renal infarction, and right popliteal artery thrombosis, which had been treated with embolectomy and anticoagulation one year prior to this presentation.

Upon triage, his vital signs were notable for a blood pressure of 92/71 mmHg, a heart rate of 110 beats/min, a respiratory rate of 18 breaths/min, a temperature of 98.3 °F, and an oxygen saturation of 98% on room air. A physical examination revealed an uncomfortable and anxious male exhibiting homonymous field loss with a more pronounced color vision deficit in the right eye. He displayed no tenderness in the temporal areas or signs of jaw claudication. A systolic murmur was heard in the mitral area with a regular rhythm. There was tenderness to palpation in the left anterior chest area, while the remainder of the physical exam was unremarkable.

Initial laboratory findings showed normocytic anemia, thrombocytopenia, elevated creatinine, and hypoalbuminemia (Table 1).

**Table 1:** Initial laboratory data ALT, alanine transaminase; AST, aspartate aminotransferase; ESR, erythrocyte sedimentation rate; LDH, lactate dehydrogenase; MCH, mean corpuscular hemoglobin; MCHC, mean corpuscular hemoglobin concentration; MCV, mean corpuscular volume

Variable	On admission	Reference range
White cell count	7.1	4.2–9.1 10*3/uL
Neutrophils (%)	73.50%	34.0–67.9%
Neutrophils (10)	1.19	1.56–6.13 10*3/uL
Lymphocytes	16.70%	21.8–53.1%
Monocytes	6.60%	5.3–12.2%
Eosinophils	2.10%	0.8–7.0%
Hemoglobin	8.9	13.7–17.5 gm/dL
Hematocrit	29.1	40.1–51.0%
Platelet count	117	150–450 10*3/uL
MCV	77.5	79.0–92.2 fL
MCH	23.8	25.7–32.2 pg
MCHC	30.7	32.3–36.5 gm/dL
Sodium	136	135–145 mEq/L
Potassium	4.1	3.5–5.3 mEq/L
Chloride	106	96–108 mEq/L
Glucose	102	70–99 mg/dL
Calcium	9.1	9.2–11.0 mg/dL
Creatinine	1.4	0.6–1.2 mg/dL
ALT	18	4–36 IU/L
AST	23	8–33 IU/L
Bilirubin total	0.2	0.1–1.2 mg/dL
Alkaline phosphatase	80	38–126 IU/L
Magnesium	1.9	1.3–2.1 mEq/L
Troponin	0.15	0.00–0.48 ng/mL
LDH	273	100–190 IU/L
ESR	32	0–20 mm/hr
C-reactive protein	5.11	0.00–1.00 mg/dL

**Figure 1:**
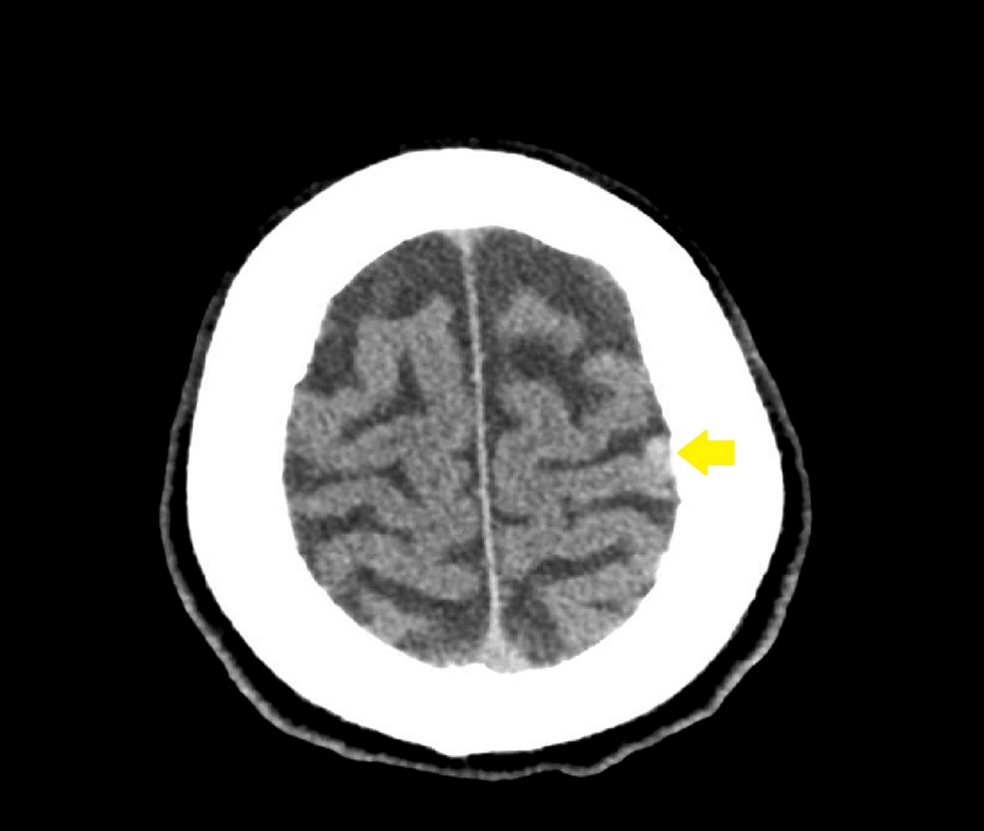
Head CT showing a new small focus of high density at the high left parietal convexity, likely representing acute intracranial hemorrhage

**Figure 2:**
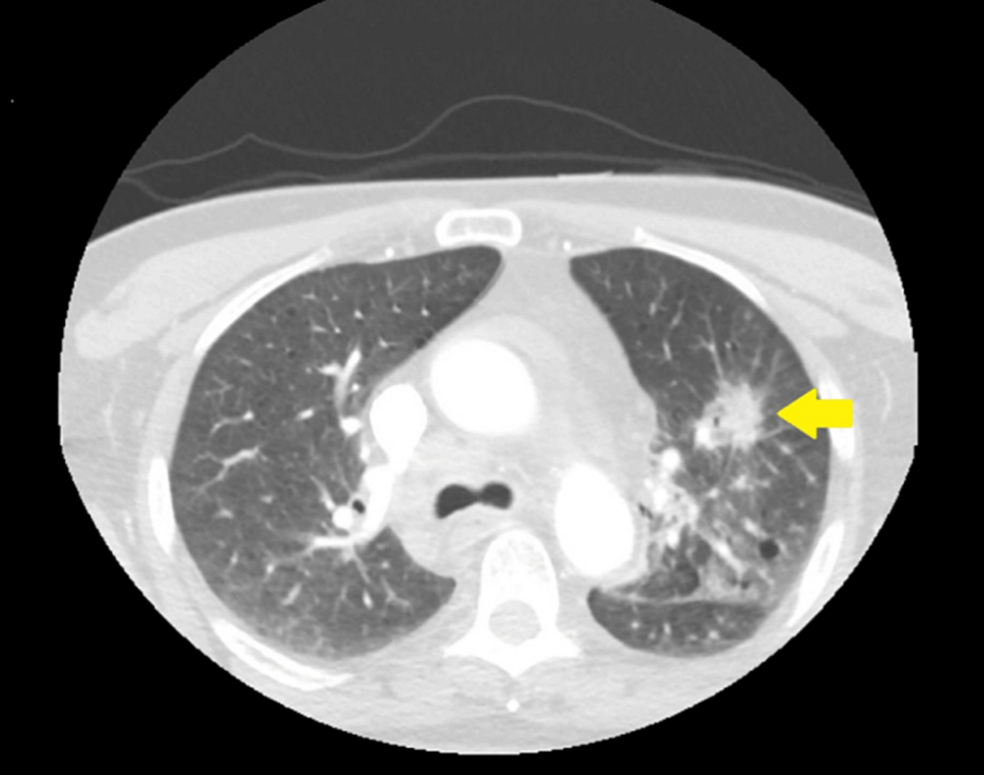
CTA of the neck showing a spiculated mass lesion affecting the left upper lobe, with adjacent patchy airspace opacities in the left mid-lung field, along with prominent mediastinal adenopathy CTA, CT angiography

The chest radiograph revealed a left perihilar hazy opacity. An electrocardiogram indicated sinus tachycardia. CT of the brain with and without contrast revealed acute cortical infarction in the left parietal and occipital lobes, along with a new small high-density focus in the high left parietal convexity, likely representing acute intracranial hemorrhage (Figure 1). The CT head angiogram did not indicate significant intracranial stenosis, and the CT neck angiogram showed no significant extracranial stenosis according to the North American Symptomatic Carotid Endarterectomy Trial criteria. However, it did reveal a spiculated mass lesion involving the left upper lobe, accompanied by prominent adjacent patchy mediastinal adenopathy (Figure 2). This lesion had been biopsied weeks earlier via endobronchial ultrasound, confirming it to be adenocarcinoma.

**Figure 3:**
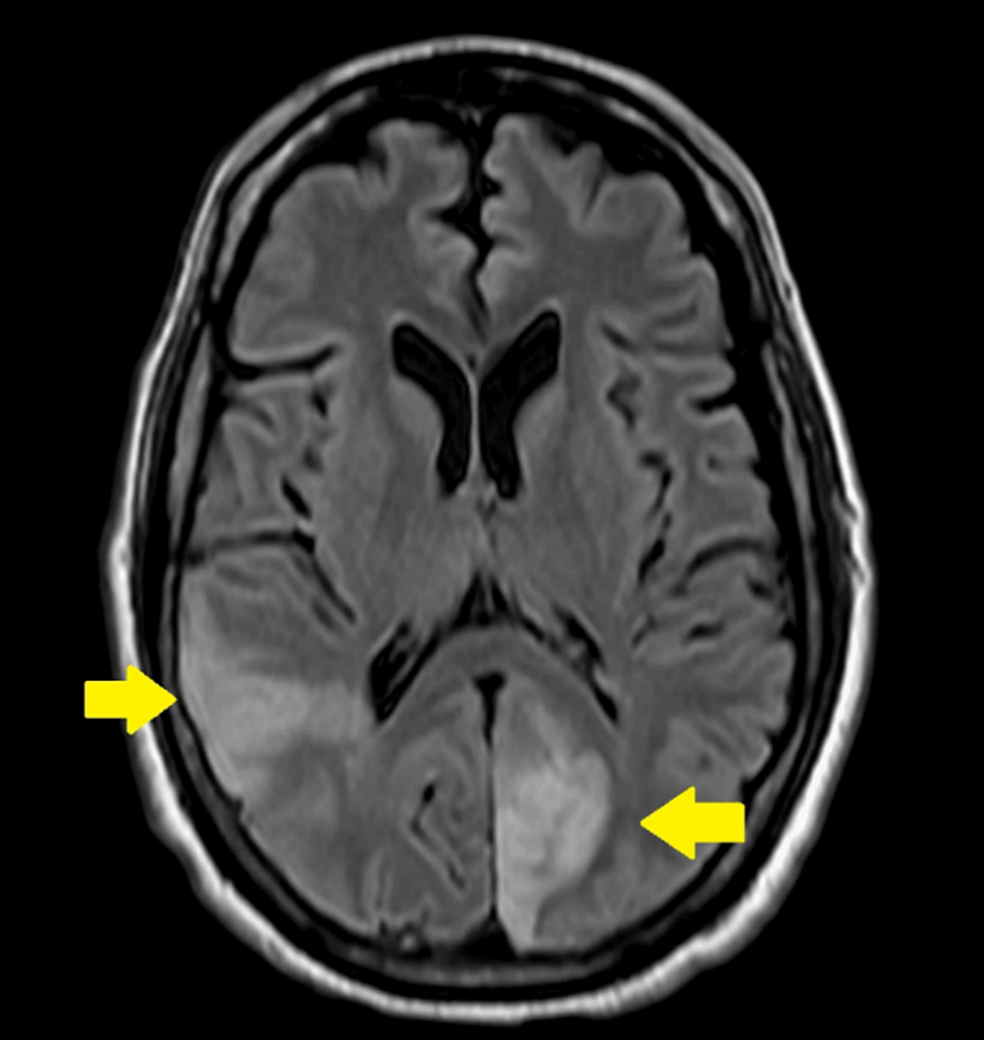
MRI brain showing acute infarcts involving the left medial occipital lobe and the right temporal occipital lobe

An MRI with and without contrast was performed 48 hours after presentation, revealing acute infarcts in the left medial occipital lobe, the right temporal occipital lobe, and scattered punctate foci in the bilateral semiovale center and corona radiata, with a predominance on the left side (Figure 3). Additionally, a small focus of intraparenchymal hematoma was observed on the high left parietal convexity, distinct from the sites of the infarcts. The echocardiogram indicated thickened mitral valve leaflets with a mass adherent to the posterior mitral leaflet, along with mild to moderate mitral valve regurgitation.

Broad-spectrum antibiotics, including vancomycin, cefepime, and doxycycline, were initiated for the patient, pending identification of a possible infection source. An extensive evaluation for infectious or hematological causes (Table 2) was conducted, with all results returning negative.

**Table 2:** Other laboratory data PCR, polymerase chain reaction

Variable	Result	Reference range
Cryptococcus antigen	Negative	Negative
Fungitell serum	<31	<80 pg/mL
Anticardiolipin antibody IgG	Negative <9	<15
Anticardiolipin antibody IgM	Negative <9	<13
Brucella IgG antibody	Negative	Negative
Brucella IgM antibody	Positive	Negative
*Bartonella henselae* IgG	Negative	<1:320
*Bartonella henselae* IgM	Negative	<1:100
Q fever antibodies IgG (Phases I and II)	Negative	<1:16
Lupus anticoagulant	Negative	Negative
Aspergillus antigen	0.02	0.00–0.29 index
HIV	Reactive	Nonreactive
CD4 count	237	359–1,519/uL
Legionella urine	Negative	Negative
Blood culture	No growth	No growth
COVID PCR test (nasopharyngeal swab)	Negative	Negative
Fibrinogen	146	200–400 mg/dL
D-dimer	>35.20	0.00–0.50 mg FEU/L

Given the clinical context of previous and current thromboembolic events, anticoagulation was deemed necessary but was delayed for 48 hours due to the discovery of a new hemorrhagic stroke upon admission. The diagnosis of marantic endocarditis was established based on negative blood cultures and the absence of evidence suggesting an infectious etiology. This conclusion was supported by the patient’s history of untreated lung adenocarcinoma with multiple embolic episodes, along with observed thrombus and fibrinous material deposition on the mitral valve. Regrettably, the patient chose to leave against medical advice after 24 hours of initiating anticoagulation and did not attend follow-up at the outpatient clinic. Nevertheless, persistently negative cultures align with the diagnosis of marantic endocarditis.

## Discussion

Marantic is a rare NIE that affects primarily the aortic and mitral valves [13,14]. It is frequently associated with immunologic disorders, advanced malignancies due to hypercoagulable states, and/or carcinomatosis [1,15]. In several studies, immunologic disorders such as SLE, BD, RA, and others have demonstrated granular deposition patterns of immunoglobulins and complement complexes along the edges of valve leaflets and within valvular vegetation. Therefore, immune complexes are also part of the pathogenic mechanism of valvular damage [16,17].

Advanced malignancies like lung and pancreatic adenocarcinomas [18] are associated with a multifactorial hypercoagulable state [19], which is a primary mechanism contributing to marantic endocarditis.

Malignancies are associated with elevated levels of circulating cytokines, such as tumor necrosis factor and IL-1, which can cause local damage to valve leaflets at the endothelium level, triggering platelet and fibrin deposition and instigating vegetation formation. However, the most critical factor in forming these vegetations may be the hypercoagulable state associated with malignancies [15,20,21].

Furthermore, animal studies showed a positive correlation between vegetation formation and increased levels of circulating tissue factor, which develops mRNA expression by valvular monocytes [21-23]. The incidence is poorly documented, especially because it is usually diagnosed postmortem. Bussani et al. analyzed more than 50,000 autopsies, and none of the NIEs were diagnosed premortem [5], further supporting this information.

The most common malignancies associated with NIE are adenocarcinomas of the lung, ovary, biliary system, pancreas, and stomach; these neoplasms are frequently mucin-secreting adenocarcinomas [20,24,25]. Despite the literature not describing pathognomonic signs and symptoms, our patient presented a dramatic clinical picture of multiple thromboembolic events due to a hypercoagulable state associated with lung malignancy, in addition to a new cardiac murmur with an intracardiac injury, raising deep concerns for nonbacterial thrombotic endocarditis (NBTE). Extensive diagnostic work excluded infectious etiology.

For patients with newly acquired murmurs, a transthoracic echocardiogram (TTE) or transesophageal echocardiogram (TEE) should be performed to identify any valvular lesion, the latter being 90% more sensitive, especially for vegetation that is <5 mm since the esophagus is adjacent to the left atrium; hence, the TEE provides better visualization of the mitral valve compared to the TTE [15].

At least three sets of blood cultures should be taken, and when the etiology is unclear, a study of hypercoagulable states should be performed. Diffusion-weighted MRI is an imaging technique that can help differentiate a stroke caused by infective endocarditis of NBTE due to the location of the stroke. The latter is multiple disseminated lesions varying in size [26].

There are no specific guidelines for treating NBTE. Management is directed toward the underlying condition, which in our case is malignancy, and toward treating thromboembolism with heparin, as per the 2008 ACCP Guidelines recommendations. This should continue indefinitely [25].

## Conclusions

Our case illustrates the complex relationship between malignancy and endocarditis and provides a scarce report of marantic endocarditis in a patient with metastatic lung adenocarcinoma. Marantic endocarditis is often secondary to or related to hypercoagulation states in patients with advanced neoplastic disease, such as adenocarcinoma of the lung or pancreas. Moreover, our case highlights the need to consider noninfectious causes of endocarditis in patients with malignancies, particularly in a hypercoagulable state and setting of thromboembolic events, to help guide prompt diagnosis and management of complex clinical presentations and optimize patient outcomes. Further research and awareness will help guide improved management in the future.
